# Mesothelioma response to carbon nanotubes is associated with an early and selective accumulation of immunosuppressive monocytic cells

**DOI:** 10.1186/s12989-016-0158-0

**Published:** 2016-08-23

**Authors:** François Huaux, Virginie d’Ursel de Bousies, Marie-Astrid Parent, Micaela Orsi, Francine Uwambayinema, Raynal Devosse, Saloua Ibouraadaten, Yousof Yakoub, Nadtha Panin, Mihaly Palmai-Pallag, Pierre van der Bruggen, Christian Bailly, Riccardo Marega, Etienne Marbaix, Dominique Lison

**Affiliations:** 1 Louvain centre for Toxicology and Applied Pharmacology (LTAP), Institut de Recherche Experimentale et Clinique (IREC), Université catholique de Louvain, Avenue Mounier 53 bte B1.52.12, 1200 Brussels, Belgium; 2 Ludwig Institute for Cancer Research, Brussels Branch, de Duve Institute, Université catholique de Louvain, Brussels, Belgium; 3 Bio and Soft Matter (BSMA), Institute of Condensed Matter and Nanosciences (IMCN), Université catholique de Louvain, Louvain-la-Neuve, Belgium; 4 Departement of Chemistry, Université de Namur, Namur, Belgium; 5 de Duve Institute, Université catholique de Louvain, Brussels, Belgium

**Keywords:** Immunosuppression, Inflammation, Mesothelioma, Myeloid cells, Carbon nanotubes, Asbestos, Crocidolite, Fibers, Rats and mice

## Abstract

**Background:**

The asbestos-like toxicity of some engineered carbon nanotubes (CNT), notably their capacity to induce mesothelioma, is a serious cause of concern for public health. Here we show that carcinogenic CNT induce an early and sustained immunosuppressive response characterized by the accumulation of monocytic Myeloid Derived Suppressor Cells (M-MDSC) that counteract effective immune surveillance of tumor cells.

**Methods:**

Wistar rats and C57BL/6 mice were intraperitoneally injected with carcinogenic multi-walled Mitsui-7 CNT (CNT-7) or crocidolite asbestos. Peritoneal mesothelioma development and immune cell accumulation were assessed until 12 months. Leukocyte sub-populations were identified by recording expression of CD11b/c and His48 by flow cytometry. The immunosuppressive activity on T lymphocytes of purified peritoneal leukocytes was assessed in a co-culture assay with activated spleen cells.

**Results:**

We demonstrate that long and short mesotheliomagenic CNT-7 injected in the peritoneal cavity of rats induced, like asbestos, an early and selective accumulation of monocytic cells (CD11b/c^int^ and His48^hi^) which possess the ability to suppress polyclonal activation of T lymphocytes and correspond to M-MDSC. Peritoneal M-MDSC persisted during the development of peritoneal mesothelioma in CNT-7-treated rats but were only transiently recruited after non-carcinogenic CNT (CNT-M, CNT-T) injection. Peritoneal M-MDSC did not accumulate in mice which are resistant to mesothelioma development.

**Conclusions:**

Our data provide new insights into the initial pathogenic events induced by CNT, adding a new component to the adverse outcome pathway leading to mesothelioma development. The specificity of the M-MDSC response after carcinogenic CNT exposure highlights the interest of this response for detecting the ability of new nanomaterials to cause cancer.

**Electronic supplementary material:**

The online version of this article (doi:10.1186/s12989-016-0158-0) contains supplementary material, which is available to authorized users.

## Background

Excessive inhalation of long and biopersistent fibrous materials such as asbestos has been associated with the development of multiple respiratory manifestations, including lung cancer and mesothelioma [1]. Concerns about similar health risks have been expressed for carbon nanotubes (CNT). The International Agency for Cancer Research (IARC) has recently recognized a specific type of CNT, i.e. Mitsui-7 CNT (CNT-7), as carcinogenic in experimental animals [2]. It is, however, not possible to generalize this carcinogenic property to other types of CNT due to the lack of coherent experimental data. There is, therefore, a need to develop bioassays and biomarkers to predict the carcinogenic activity of existing, new or emerging CNT.

The currently prevailing paradigm accounting for the pathogenic activity of carcinogenic fibers such as asbestos or CNT is relatively simple: a chronic inflammatory status, in which immune cells are activated and release toxic mediators, damages the pulmonary architecture, modulates the accumulation of mesenchymal cells and their connective tissue products, and transforms lung epithelial or mesothelial cells through direct and indirect genotoxic and epigenetic pathways [3]. Experimental monitoring of this inflammatory response can be used to predict the carcinogenic activity of CNT in short-term bioassays. In their seminal work, Poland and colleagues revealed the potential of long straight CNT to induce mesothelioma by documenting their capacity to elicit an inflammatory response, similar to asbestos fibres, in the mouse peritoneal cavity [4].

There is now growing evidence that, besides inflammation, immunosuppressive responses also contribute to the pathological processes involved in carcinogenesis and cancer progression [5, 6]. Tumors harbor immunosuppressive cells (i.e. regulatory macrophages, monocytes, DC, neutrophils and lymphocytes) that inhibit both innate and adaptive immunity, subverting immune surveillance and preventing efficient natural or therapeutic anti-tumor immune responses [7]. The current understanding of immunosuppression in malignant mesothelioma has advanced significantly within the past decade [8]. Tumor-associated immunosuppressive cells include T regulatory lymphocytes (T regs) and tumor-associated macrophages (M2 macrophages). Indeed, the stromal space in primary human mesothelioma tissue harbors significant proportions of immunosuppressive T regs and M2 macrophages that suppress adaptive anti-tumor immunity in this disease [9, 10].

Myeloid-derived suppressor cells (MDSC) include a small group of discrete myeloid progenitors and immature cells morphologically and phenotypically similar to monocytes (M-MDSC) or polymorphonuclear cells (PMN-MDSC) [11]. MDSC are currently the focus of intense research efforts as their accumulation in neoplastic tissues represents a prognostic marker of tumor progression and poor therapeutic efficiency [12]. MDSC are generally associated with established neoplastic tissue, their recruitment being induced by tumor-derived factors [11, 12]. Treatment of mesothelioma-bearing mice with the COX-2 inhibitor celecoxib prevented the local and systemic expansion of MDSC, suggesting that prostaglandins such as PGE2 play an important role in the recruitment of MDSC in established mesothelioma [13]. Immunotherapeutic approaches targeting MDSC or their immunosuppressive function have recently been suggested in patients with mesothelioma [8].

The direct exposure of mesothelial cells to particles in the rat peritoneum bioassay is very useful to identify the capacity of fibrous materials to induce mesotheliomas [4, 14–16]. While this model does not cover all the aspects of what occurs in humans occupationally or environmentally exposed, it allows easily sampling the mesothelial cavity for studying immune cells associated with mesotheliomagenesis [4]. We used this model and a panel of CNT previously characterized for their capacity to induce or not experimental mesothelioma [17–19] to define the inflammatory and immunosuppressive functions of accumulated leucocytes after CNT exposure. We newly show here that M-MDSC are selectively recruited during the early peritoneal response to carcinogenic CNT, well before the establishment of mesothelioma.

## Results

### Monocytic MDSC accompany CNT-induced mesotheliomas in rats

**Fig. 1:**
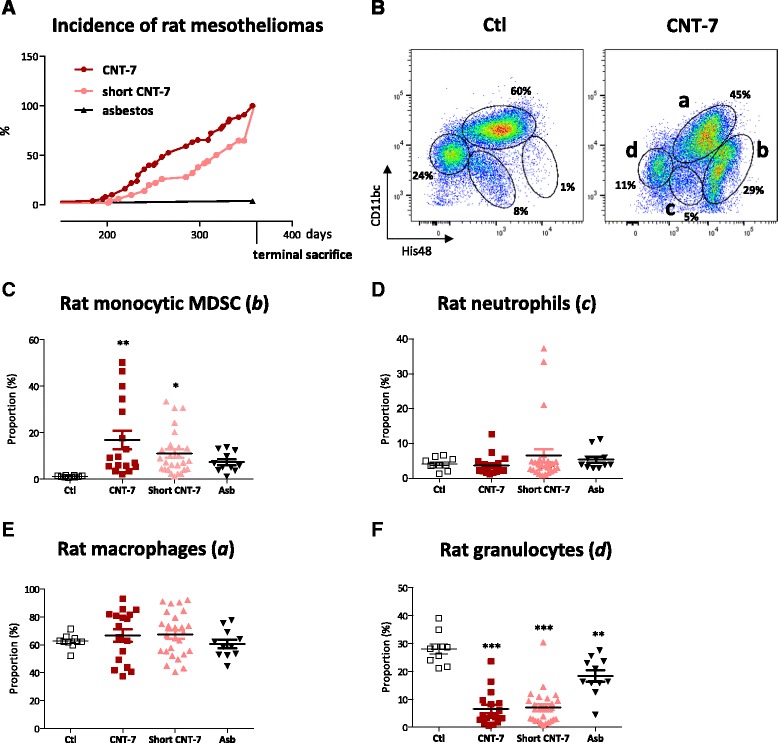
M-MDSC are specifically accumulated in the peritoneal cavity of rats bearing mesotheliomas after carcinogenic CNT-7 exposure. Incidence of peritoneal mesotheliomas (**a**). Flow cytometry dot plots show the proportions of peritoneal leukocyte sub-populations (**b**) present in the peritoneal cavity of Wistar rats bearing or not mesotheliomas after carcinogenic CNT-7 (6 mg) or saline (Ctl) treatments and identified by using CD11b/c and His48 markers (a = macrophages, b = M-MDSC, c = neutrophils, d = granulocytes, i.e. eosinophils and mast cells, as indicated in CNT-7 square)**.** Proportion of (**c**) monocytic CD11b/c^int^ and His48^high^ Myeloid Derived Suppressor Cells (M-MDSC) (see *b* in B), (**d**) CD11b/c^int^ and His48^int^ neutrophils (see *c* in B), (**e**) CD11b/c^high^ and His48^int^ macrophages (see *a* in B), and (**f**) CD11b/c^int^ and His48^low^ granulocytic cells (see *d* in B, eosinophils-mast cells) in peritoneal fluid after saline, CNT-7, short CNT-7 (6 mg) or asbestos (Asb, 2 mg) intra-peritoneally injected in rats. Each bar represents mean ± SEM. The results were statistically analyzed using the Dunnett’s Multiple Comparison test. ** = *p* < 0.01, *** = *p* < 0.001 indicate a statistically significant difference with saline-treated rats (Ctl)

We first traced MDSC in the peritoneal cavity of rats treated with carcinogenic multi-walled Mitsui-7 CNT (CNT-7) known to induce mesotheliomas in Wistar rats, a strain susceptible to carcinogenic fibers [17–19]. Rats were treated with a single intra-peritoneal injection of CNT-7 (6 mg, median length, 7.1 μm; 78 % >5 μm), short CNT-7 (6 mg, median length, 2.8 μm; 14 % > 5 μm) or crocidolite asbestos [17]. As expected, the first mesotheliomas occurred 6 months after CNT-7, the majority of animals developing tumors within 12 months (Fig. 1a). Short CNT-7 also induced mesotheliomas although to a lesser extent than the long ones (Fig. 1a). Mesothelioma diagnosis was confirmed by histological analyses (Additional file 1: Figure S1 A-F). CNT-7 and short CNT-7 appeared extremely active in comparison to asbestos fibers over the 12-month duration period (Fig. 1a). One rat injected with asbestos developed a mesothelioma. This low response to asbestos after 12 months was consistent with our previous work in the same rat strain in which the same asbestos sample did not induce mesothelioma until 14 months after injection [19]. None of the controls developed mesothelioma. Leucocytes were detected in the peritoneal lavage performed when rats were euthanized in the course of the experiment because of clinical evidence of mesothelioma (mainly presenting with a dilated abdomen and ascites) or at terminal sacrifice for rats injected with CNT-7 or asbestos. Leukocytes were identified by recording expression of CD11b/c and His48 by flow cytometry [20, 21]. Monocytic cells and macrophages were the two main populations present in the peritoneal cavity of rats bearing mesothelioma after CNT treatment (Additional file 1: Figure S1 G and I). Compared to control rats, increased proportions of monocytic CD11b/c^int^His48^high^ cells were noted in animals injected with CNT-7 (Fig. 1b-c), short CNT-7 or asbestos (Fig. 1 c and Additional file 1: Figure S1 H), and the extent of their accumulation was associated with the ability of the fibers to induce mesotheliomas over the 12 months observation period (Fig. 1a). In contrast, mesotheliomas were not associated with an increased proportion of neutrophils (CD11b/c^int^ and His48^int^) (Fig. 1d) and macrophages (CD11b/c^high^ and His48^int^) (Fig. 1e). The proportion of granulocytic cells (eosinophils-mast cells, CD11b/c^int^ and His48^low^) was significantly decreased upon mesotheliomagenic particle exposure (Fig. 1f). We thus concluded that M-MDSC specifically accumulated in the peritoneal cavity of rats bearing mesotheliomas induced by CNT.

### Early accumulation of M-MDSC in response to carcinogenic CNT

**Fig. 2:**
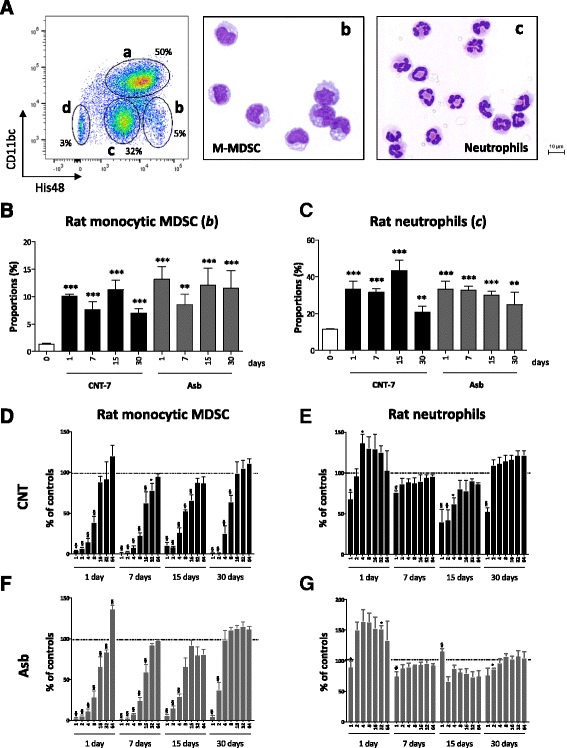
An early accumulation of immunosuppressive M-MDSC is specifically associated with the peritoneal response to carcinogenic CNT-7 in Wistar rats. **a** Flow cytometry dot plots show the proportions of leukocyte sub-populations present in peritoneal lavages obtained from CNT-7-treated rats (2 mg) at day 7 and identified by using CD11b/c and His48 markers (a = macrophages, b = M-MDSC, c = neutrophils, d = granulocytes, i.e. eosinophils and mast cells). Microscopic views (Diff-Quick staining, 400x) of FACS-sorted monocytic CD11b/c^int^ and His48^hi^ Myeloid Derived Suppressor Cells (M- MDSC) (b) and CD11b/c^int^ and His48^int^ neutrophils (c) freshly obtained from peritoneal cells of CNT-7-treated rats at day 7. Proportion of (**b**) M-MDSC and (**c**) neutrophils in the peritoneal lavages performed on CNT-7- or crocidolite asbestos- (Asb) treated Wistar rats at different time points (from 1 to 30 days) after ip injection of 2 mg particles. Each bar represents mean ± SEM of 4–5 observations. The results were statistically analyzed using the Dunnett’s Multiple Comparison test. ** = *p* < 0.01, *** = *p* < 0.001 indicate a statistically significant difference with saline-treated rats (day 0). In vitro anti-proliferative activity of different numbers of M-MDSC (**d**-**f**) or neutrophils (**e**-**g**) purified at different time points (from 1 to 30 days) from CNT-7- (**d**-**e**) or asbestos- (**f**-**g**) treated rats (2 mg) on spleen T lymphocytes activated with anti-CD3/CD28 antibodies. Graphs represent the average level of ^3^H-thymidine incorporation expressed as percent of controls (activated T lymphocytes alone) for each M-MDSC or neutrophils/splenocytes ratio. Each bar represents mean ± SEM. The results were statistically analyzed using the Dunnett’s Multiple Comparison test. * = *p* < 0.05, $ = *p* < 0.01, # = *p* < 0.001 indicate a statistically significant difference with activated splenocytes cultured alone

We next determined the early response to CNT by examining the time course of peritoneal leukocyte influx from day 1 to 30. CNT and asbestos (2 mg) induced an acute accumulation of peritoneal leukocytes after injection (Additional file 1: Figure S2 A). We found that monocytic CD11b/c^int^ His48^high^ cells appeared in the peritoneal cavity as early as 24 h after injection and persisted for at least 30 days (Fig. 2a-b). Monocytic cells purified from 1 to 30 days after CNT displayed a strong immunosuppressive activity on spleen T lymphocytes activated with anti-CD3/CD28 antibodies (Fig. 2d), confirming that this cell population comprised M-MDSC. In contrast, monocytes purified from blood or peritoneal cavity of naive rats did not significantly affect activated T cell proliferation in co-cultures (Additional file 1: Figure S4 A). A neutrophilic CD11b/c^int^ His48^int^ cell population was also recruited at the early stages after CNT administration (Fig. 2a and c) but these cells did not display immunosuppressive activity (Fig. 2e), indicating that they consisted of inflammatory neutrophils. Like CNT, asbestos strikingly induced M-MDSC and inflammatory neutrophil accumulation at all-time points examined (Fig. 2b-c and f-g). Finally, the proportions of regulatory macrophages and other inflammatory granulocytes (eosinophils and mast cells) were decreased by carcinogenic fiber treatment (Additional file 1: Figure S3).

We next demonstrated that infiltrated M-MDSC proliferate as appreciated by BrdU incorporation (Additional file 1: Figure S4 B) and weakly expressed the monocyte/macrophage lineage markers CD68 and CD163 [22] (Additional file 1: Figure S4 C-D). These observations supported the notion that monocytic cells recruited after CNT-7 exposure were undifferentiated and proliferating cells and corresponded to bona fide MDSC. Finally, we confirmed the inflammatory profile of neutrophilic cells in CNT-7-treated rats by revealing that these cells weakly expressed Rp-1 (Additional file 1: Figure S4 E), a marker of PMN-MDSC in rat mammary carcinoma [21]. Altogether, these results indicated that, besides the inflammatory reaction (inflammatory neutrophils), an early accumulation M-MDSC was selectively associated with the peritoneal response to carcinogenic CNT.

### Early peritoneal M-MDSC response appears specific for mesotheliomagenic CNT

We next determined whether M-MDSC were recruited in mice, which do not develop mesothelioma upon intra-peritoneal injection of CNT or asbestos fibers [23–25]. In our hands, conventional C57BL/6 mice did not develop mesothelioma even after multiple injections of CNT-7 (0.2 mg/mouse/week, not shown). The kinetics of total leukocyte recruitment in the peritoneal cavity of CNT- and asbestos-treated mice was similar to that recorded in rats (Additional file 1: Figure S2 B). The leukocyte subpopulations were identified in mice by using CCR2 and Ly6G markers [11]. We found that while the proportions of monocytic and neutrophilic cells were increased after CNT-7 and asbestos injections (Additional file 1: Figure S5 A-C), these cells did not possess a significant and persistent immunosuppressive activity (Additional file 1: Figure S5 D-G). Macrophages and granulocytes were not accumulated after particle treatment and did not affect T cell proliferation in vitro (Additional file 1: Figure S6). These data indicated that only inflammatory leukocytes are recruited in CNT-treated mice. Combining observations in rats and mice supported, therefore, the idea that the development of peritoneal mesotheliomas after CNT exposure is related to an early recruitment of both inflammatory neutrophils and M-MDSC.

**Fig. 3:**
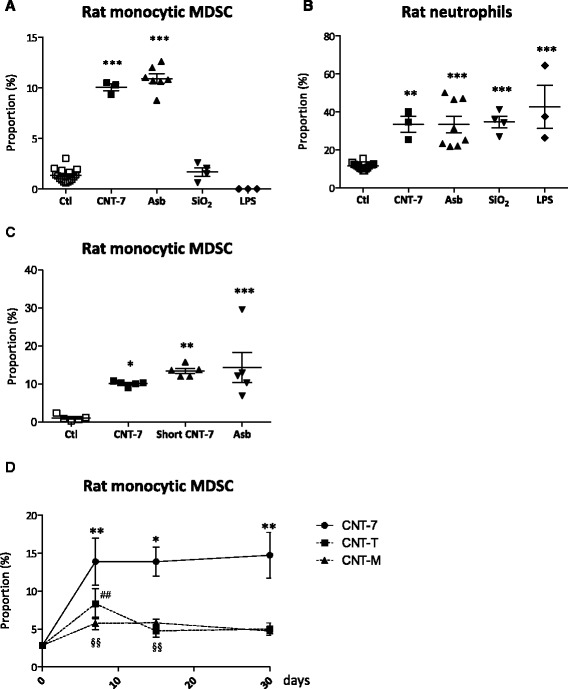
The early peritoneal M-MDSC response is specific of mesotheliomagenic CNT. Proportion of (**a**) monocytic CD11b/c^int^ and His48^hi^ Myeloid Derived Suppressor Cells (M-MDSC) and (**b**) inflammatory CD11b/c^int^ and His48^int^ neutrophils in the peritoneal lavages performed on saline-, CNT-7- (2 mg), crocidolite asbestos- (Asb, 2 mg), DQ12 silica- (SiO_2_, 2 mg) or LPS- (E. coli, 5 μg) treated Wistar rats 1 day after ip injection. SiO_2_ and LPS are not mesotheliomagenic. **c** Proportion of peritoneal M-MDSC after ip injection of 2 mg CNT-7, short CNT-7 or asbestos at day 1. Each bar represents mean ± SEM of 4–5 observations. The results were statistically analyzed using the Dunnett’s Multiple Comparison test. * = *p* < 0.05, ** = *p* < 0.01, *** = *p* < 0.001 indicate a statistically significant difference with saline-treated rats (Ctl). **d** Proportion of peritoneal M-MDSC in the peritoneal cavity of rats after carcinogenic CNT-7 or non-carcinogenic CNT-M or CNT-T treatment (2 mg) at days 7, 15 and 30. The results were statistically analyzed using the Dunnett’s Multiple Comparison test. * = *p* < 0.05, ** = *p* < 0.01, *** = *p* < 0.001 indicate a statistically significant difference between saline-treated (day 0) and CNT-7-treated animals. ## = *p* < 0.01 indicates a statistically significant difference between saline-treated (day 0) and CNT-T-treated animals. $$ = *p* < 0.01 indicates a statistically significant difference between saline-treated (day 0) and CNT-M-treated animals

We further evaluated the specificity of the early M-MDSC accumulation by comparing their influx at early time points (days 1 and 7) after a single injection of different agents inducing mesotheliomas (CNT-7, asbestos, 2 mg) or not (silica 2 mg, LPS dose 5 μg) in Wistar rats. We recorded an inflammatory reaction in all groups as illustrated by the accumulation of inflammatory neutrophils. However, an accumulation of M-MDSC was only evident in animals treated with CNT-7 or asbestos (Fig. 3a-c and Additional file 1: Figure S7).

We also compared the capacity of CNT-7 and non-carcinogenic CNT (CNT-M [19] and CNT-T [17]) at a dose of 2 mg to acutely recruit M-MDSC in the peritoneal cavity of Wistar rats. Only CNT-7 induced an early and persistent accumulation of M-MDSC while this influx was weak and transient after CNT-M and CNT-T (Fig. 3d and Additional file 1: Figure S7). Altogether, an early and sustained peritoneal M-MDSC response appeared specific for a mesotheliomagenic CNT.

## Discussion

While the mechanistic evidence for a mesotheliomagenic activity of CNT is still limited, recent experimental studies have shown that CNT-7 (like asbestos) induce peritoneal mesothelioma in rats [17, 18, 26]. These data are challenging because, in humans, malignant mesothelioma remains highly refractory to existing therapies [27]. There is, therefore, an urgent need for additional investigations to identify pathological pathways implicated in CNT-induced mesotheliomas and new biomarkers predicting the carcinogenic activity of newly developed CNT.

Major advances in understanding the mechanisms leading to tumor immunity have recently been made [28–30]. The development of cancers is now viewed as related to the ability of tumors to develop a tolerant microenvironment by activating diverse immunosuppressive mechanisms counteracting antitumor host immunity [31]. Previous studies have indicated that immunosuppressive responses in mesothelioma consist of tumor-induced accumulation of regulatory T lymphocytes (Tregs) and immature myeloid cells [32]. By using orthotopic implantation of established mesothelioma cell lines in syngenic mice, it has been reported that tumor-associated Tregs contribute to mesothelioma development by limiting the accumulation of IFN-gamma producing effector T lymphocytes [9, 33]. COX-2-dependent MDSC have also been reported in established mesothelioma [13, 34]. In a mouse model of lung carcinoma, CNT increased the recruitment and accumulation of tumor-associated MDSC that produced TGFβ and increased lung tumor burden [35, 36]. Our data demonstrate for the first time that carcinogenic CNT possess the intrinsic capacity to induce a selective, rapid and sustained accumulation of monocytic MDSC before mesothelioma is established. This unsuspected observation indicates that a suitable environment for tumoral cell evasion from T cell surveillance is rapidly installed (within the first days) after carcinogenic CNT exposure. M-MDSC can be generated in the bone marrow in response to factors such as colony stimulating factors (CSFs), inflammatory cytokines and vascular endothelial growth factors and are recruited through a gradient of chemokines such as CCL2. Resident peritoneal macrophages and mesothelial cells treated in vitro with asbestos or CNT release these MDSC-related factors [37, 38]. Our data also revealed that beside monocytic cells, peritoneal macrophages also possess immunosuppressive functions upon CNT treatment (Additional file 1: Figure S3). The immunosuppressive M-MDSC and macrophages may thus represent a new crucial pathological pathway in CNT-induced mesothelioma. Our observation also showed that M-MDSC weakly expressed the markers of the classical monocyte/macrophage lineage CD68 and CD163. Previous study also demonstrated that tumor-associated macrophages are not related to MDSC in experimental mesothelioma [39]. Altogether, these data suggest that monocytic cells weakly differentiate in macrophages and that other mechanisms explain macrophage accumulation during peritoneal responses to CNT. Interestingly, it has been demonstrated that macrophages self-maintain independently of monocytes in response to IL-1α released after silica instillation [40] and hypothetically after intra-peritoneal administration of CNT. Regulatory neutrophilic MDSC (PMN-MDSC) that highly express Rp-1, a marker of undifferentiated neutrophils, have been detected in rat bearing carcinoma [21]. In contrast, neutrophils accumulated locally after short-term exposure to mesotheliomagenic CNT are ineffective for inhibiting T cell proliferation and weakly express Rp-1. It remains, however, to determine whether regulatory Rp-1^+^ PMN-MDSC infiltrate mesotheliomas in long-term studies with CNT-treated rats as proposed in other models [41].

In recent years, experimental observations have supported a scenario whereby a series of chronic and diverse cell injuries and immune stimuli explain the long latency period of mesothelioma development. It thus is probable that the persistent accumulation of M-MDSC represents one of the immune components contributing to mesothelioma development. Indeed, considerable evidence also suggests that inflammatory neutrophils play a critical role in cancer progression. Early in the neoplastic process, neutrophils trigger genomic instability, produce a favorable environment for tumor growth, and promote angiogenesis [42]. Neutrophils accumulated after asbestos generate substantial amounts of DNA damaging free-radicals [43]. The neutrophil influx in the peritoneal cavity of CNT-treated animals therefore contributes to explain the carcinogenicity of CNT and has been proposed as an early biomarker for predicting the carcinogenic activity of CNT [3, 4]. We confirmed here that inflammatory neutrophils are massively and early recruited after CNT or asbestos injection. The joint presence of immunosuppressive M-MDSC and inflammatory neutrophils supports a scenario in which synchronized immunosuppressive and inflammatory mechanisms contribute to the emergence of neoplastic cells, induce immunological tolerance allowing tumor cell evasion, and finally promote tumor progression. In contrast to M-MDSC, we noted that the accumulation of inflammatory neutrophils was not specific for mesotheliomagenic agents because silica or LPS similarly induced an acute accumulation of neutrophils. Thus, the early and sustained accumulation of M-MDSC after carcinogenic CNT treatment may represent a more robust indicator of a potential to induce mesothelioma than solely the inflammatory neutrophil response. The capacity of a given CNT to maintain a sustained M-MDSC response probably integrates its biopersistence, which is an important parameter determining the carcinogenic potential of CNT and fibers [3].

## Conclusion

Our data provide new insight on how CNT may establish a carcinogenic response by mounting an early and sustained immunosuppressive microenvironment facilitating, in conjunction with inflammation, mesothelioma development. M-MDSC represent a new component of the adverse outcome pathway leading to the development of mesothelioma. The specificity of the M-MDSC accumulation after exposure to carcinogenic CNT highlights the potential interest of this response for detecting in short-term bioassays the ability of new or emerging CNT to cause mesothelioma.

## Methods

### Carbon nanotubes and asbestos fibers

CNT-7 were multi-walled carbon nanotubes MWCNT-XNRI-7 from Mitsui & co (Ltd., Tokyo, Japan, Lot# 05072001 K28) sub-sampled at Norwegian Research Centre for the Working Environment (NRCWE) with the code NRCWE-006. Short CNT-7 were obtained by grinding CNT-7 in an oscillatory agate ball mill (Pulverisette 0, Fritsch), with a vertical vibration of 1 mm applied during 6 h. CNT-7 and short CNT-7 were annealed at 1500 °C under argon during 60 min. Multi-walled CNT-M (Muller) and CNT-T (tangled, kindly provided by prof S. Toyokuni, Nagoya university, Japan) and their physico-chemical characteristics have been documented in details previously [17, 19, 44]. The main characteristics of CNT-7, short CNT-7 and CNT-M are summarized in the Additional file 2: Table S1. Respirable crocidolite fibers (positive control) were obtained from the Union internationale contre le Cancer (UICC, Geneva, Switzerland) [45]. All particles were first treated at 200 °C during 2 h to remove any possible trace of endotoxin and suspensions were prepared immediately before administration by sonication and manual vortexing in sterile phosphate-buffered saline (PBS) containing 1.4 mg bovine serum albumin/ml.

#### Animals

Specific pathogen free (SPF) male Wistar rats (10 weeks old, 200 g) were obtained from Charles River (Brussels, Belgium). C57BL/6 mice (10 weeks old, 20 g) were purchased from Janvier SAS (St Berthevin, France). International recommendations for doses administered, controls and group size were followed [46]. After a week of acclimatization, the animals were injected intraperitoneally with the different particles suspended in a volume of 2 ml (rats) or 0.5 ml (mice) of PBS-BSA. For long-term experiments (12 months), groups of 50 rats were injected with 6 mg of CNT-7 (2*10^9^ WHO fibres), short CNT-7 (0.36*10^9^ WHO fibres). Positive controls were injected with 2 mg (6*10^9^ WHO fibres) of crocidolite asbestos (*n* = 26). Vehicle controls (*n* = 26) were injected with an equal volume of PBS-BSA. The animals were housed at the local animal facility under SPF conditions, with a 12-h light–dark cycle and controlled temperature and humidity. They were given tap water and sterilized pellets ad libitum. The animals were observed daily for mortality/morbidity and debilitated animals were euthanized with an intra-muscular injection of pentobarbital. Full gross necropsy was performed on all animals found dead or euthanized during the experiment, and grossly visible lesions or tumors were preserved in 10 % formalin. The experiment was terminated after 12 months when the majority of the animals injected with CNT-7 or short CNT-7 had developed tumors. At this point in time, positive control animals were not justified anymore and we sacrificed the rats treated with crocidolite before they had developed tumors, except one. All animals were sacrificed and systematically autopsied. External lesions and tumors were recorded and samples were preserved for subsequent histopathological analysis. The peritoneal cavity was opened and the cells present in the peritoneal fluid were collected and analyzed (see below). Then, the peritoneal cavity was examined macroscopically for the presence of anomalies and tissues were preserved in formalin for histopathological examination.

In short-term experiments to analyze cellular responses, groups of 4 to 5 Wistar rats or C57BL/6 mice were injected with 2 mg (rats) or 0.2 mg (mice) of test material and sacrificed after 1, 7, 15 and 30 days. In these conditions, injection in rats of 2 mg CNT-7 corresponds to 0.67*10^9^ WHO fibres, 2 mg short CNT-7 to 0.12*10^9^ WHO fibres and 2 mg crocidolite asbestos to 6*10^9^ WHO fibres. In mice, 0.67 10^8^ WHO fibres of CNT-7 (0.2 mg) were injected per animal.

#### Histopathological analysis

The different tissues were processed for light microscopic examination by serial desiccation in ethanol and paraffin embedded specimens were cut in 5-μm slices and stained with hematoxylin-eosin (HE). The preparations were examined by an experienced pathologist.

#### Cell analysis and purification

The peritoneal cavity was lavaged with 15 ml (rats) or 8 ml (mice) of saline, and leukocyte counts were determined on a Burker cell chamber. Cytocentrifuge preparations of peritoneal cells were stained with Diff-Quick (Baxter, Lessines, Belgium). Rat peritoneal cell suspensions were also stained using antibodies specific for CD11b/c-APC (clone OX-42) obtained from BioLegend (San Diego, USA), His48-FITC (clone anti-granulocytes) from eBioscience (Hatfield, UK), CCR2-PE (clone 890231) from R&D system (Abingdon, UK), CD68 and CD163 (AbD Serotec, Oxford, UK), Rp-1 (BD Pharmingen, Erembodegem, Belgium) and isotype controls (clone MOPC-21, MCA1209F, 11E10 and MG2a) from BioLegend, Gentaur (Kampenhout, Belgium), eBioscience and Invitrogen (Frederick, USA). Mouse peritoneal cells were stained by the following antibodies: CD45-PerCp Cy5.5 (clone 30-F11), CD11b-BV 421 (clone M1/70), Ly6G-FITC (clone 1A8) from BD Biosciences (Erembodegem, Belgium), CCR2-PE (clone 475301) from R&D Systems and isotype controls (clone R35-95, A95-1) from BD Biosciences. Samples were fixed in a 1.25 % paraformaldehyde in PBS, acquired on a FACSCalibur (BD Biosciences) and analyzed using the FlowJo software. Rat and mouse peritoneal leukocytes were separated and isolated using flow cytometry cell sorting (FACSAria III, BD Biosciences). APC (CD11b/c), FITC (His48) and PE (CCR2) fluorescence measured by FACS was used to select rat peritoneal cell subpopulations and PerCP-Cy5.5 (CD45), BV 421 (CD11b), FITC (Ly6G), and PE (CCR2) fluorescence to select mouse peritoneal cell subpopulations The purity of the obtained leukocyte cell preparations was routinely >93 % as assessed by Diff-Quick staining.

#### BrdU incorporation

15 mg of BrdU (BD Biosciences) was injected intraperitoneally in rats 24 h before the sacrifice (7 days after saline or CNT-7 treatment, 2 mg). Cell suspensions from the peritoneal cavity were stained for surface markers followed by intracellular BrdU staining using the BrdU Flow kit according to the manufacturer’s instructions (BD Biosciences). Stained cells were acquired on a FACSCalibur (BD Biosciences) and analyzed using the CellQuest software (BD Biosciences).

#### Immunosuppressive in vitro test

The suppressive activity on T lymphocytes of peritoneal leukocyte subpopulations was assessed in a co-culture assay in which splenocytes from 3 naive rats or mice were pooled and seeded in triplicates in 96-well round bottom plates (50× 10^3^/well, Cellstar®, 96 well, U-Botto, Greiner Bio-One, Wemmel, Belgium). Splenocytes were cultured in the presence of increasing ratios of rat or mouse purified peritoneal cells and stimulated with anti-CD3 (anti-rat clone G4.18, anti-mouse clone 145-2C11, 5 μg/ml, BD Biosciences) and anti-CD28 (anti-rat clone JJ319, anti-mouse clone 37.51, 2 μg/ml, BD Biosciences) antibodies. On day 3 of the co-culture, cells were pulsed with ^3^[H]-thymidine (0.05 mCi per well, Perkin Elmer, Waltham, USA) and 18 h later ^3^[H]-thymidine incorporation was assessed.

#### Statistics

Data were evaluated by one-way analysis of variance (ANOVA) using the Dunnett’s Multiple Comparison Test when appropriate. Statistical significance was considered at *p* < 0.05.

## Additional files

Additional file 1: Figure S1. Mesotheliomas and leucocytes in peritoneal cavity of Wistar rats after mesotheliomagenic CNT-7 exposure. **Figure S2.** Peritoneal cell numbers after CNT and asbestos treatment in rats and mice. **Figure S3.** Proportions and in vitro activity of macrophages and granulocytes during the early peritoneal response to carcinogenic CNT-7 in Wistar rats. **Figure S4.** Characterization of peritoneal M-MDSC and inflammatory neutrophils accumulated after CNT-7 injection in Wistar rats. **Figure S5.** Accumulation of inflammatory monocytes and neutrophils during the peritoneal response to carcinogenic CNT-7 in C57BL/6 mice. **Figure S6.** Proportions and in vitro activity of macrophages and granulocytes during the early peritoneal response to carcinogenic CNT-7 in C57BL/6 mice. **Figure S7.** Early peritoneal M-MDSC response is specific of mesotheliomagenic CNT in Wistar rats. (PPTX 5835 kb)

Additional file 2: Table S1. Characteristics of the carbon nanotubes. (DOCX 200 kb)

## Abbreviations

CNT: Carbon nanotubes
CNT-7: Multi-walled Mitsui-7 CNT
CNT-M: Muller carbon nanotubes
CNT-T: Tangled carbon nanotubes
Asb: Crocidolite asbestos
SiO_2_: DQ12 silica
MDSC: Myeloid-derived suppressor cells
M-MDSC: Monocytic myeloid-derived suppressor cells
PMN-MDSC: Polymorphonuclear myeloid-derived suppressor cells
