# Supplementary material for: Mesothelioma response to carbon nanotubes is associated with an early and selective accumulation of immunosuppressive monocytic cells
Source: Part Fibre Toxicol. 2016 Aug 23;13:46. doi: 10.1186/s12989-016-0158-0 (PMC4994252; doi:10.1186/s12989-016-0158-0)
Supplement: Supplementary file 1 — Mesotheliomas and leucocytes in peritoneal cavity of Wistar rats after mesotheliomagenic CNT-7 exposure. Figure S2. Peritoneal cell numbers after CNT and asbestos treatment in rats and mice. Figure S3. Proportions and in vitro activity of macrophages and granulocytes during the early peritoneal response to carcinogenic CNT-7 in Wistar rats. Figure S4. Characterization of peritoneal M-MDSC and inflammatory neutrophils accumulated after CNT-7 injection in Wistar rats. Figure S5. Accumulation of inflammatory monocytes and neutrophils during the peritoneal response to carcinogenic CNT-7 in C57BL/6 mice. Figure S6. Proportions and in vitro activity of macrophages and granulocytes during the early peritoneal response to carcinogenic CNT-7 in C57BL/6 mice. Figure S7. Early peritoneal M-MDSC response is specific of mesotheliomagenic CNT in Wistar rats. (PPTX 5835 kb) [file 12989_2016_158_MOESM1_ESM.pptx]

## Slide 1
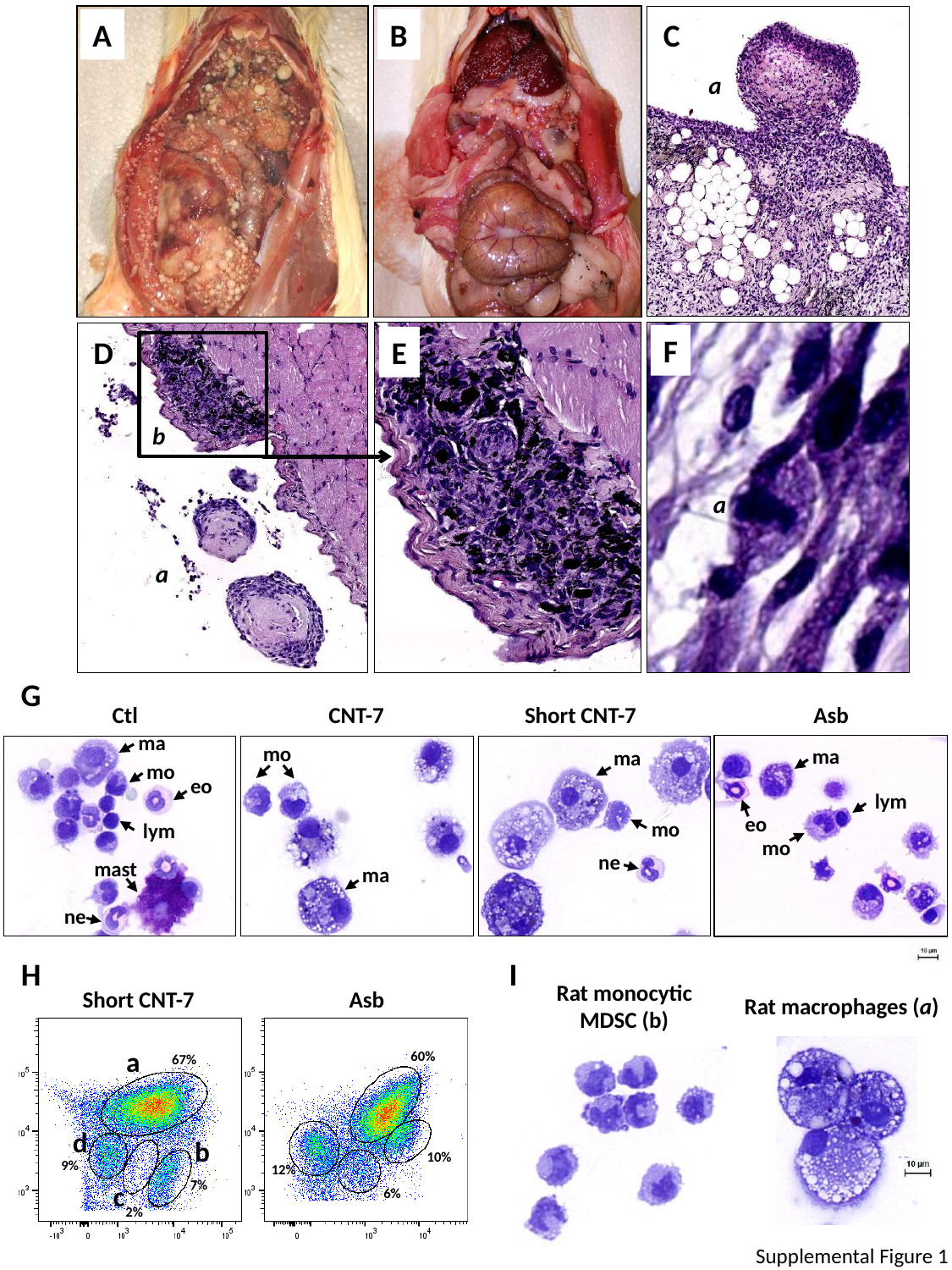

C
A
B
a
F
D
E
b
a
a
G
Ctl
CNT-7
Short CNT-7
Asb
ma
mo
ma
ma
mo
eo
lym
eo
mo
lym
mo
ne
mast
ma
ne
H
I
Rat monocytic
MDSC (b)
Short CNT-7
Asb
Rat macrophages (a)
a
60%
67%
d
b
10%
9%
12%
7%
c
6%
2%
Supplemental Figure 1

## Slide 2
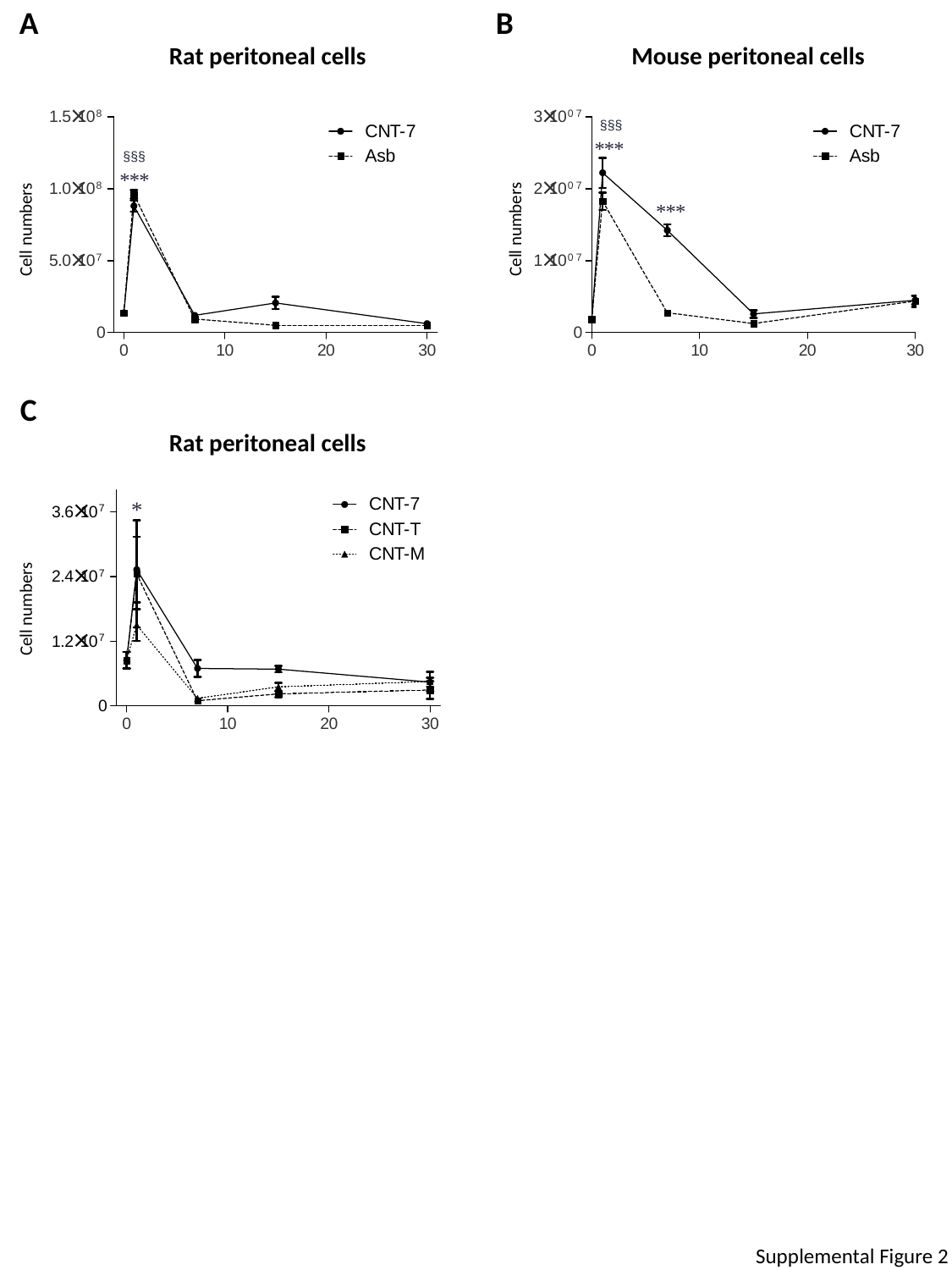

B
A
Rat peritoneal cells
Mouse peritoneal cells
Cell numbers
Cell numbers
C
Rat peritoneal cells
Cell numbers
Supplemental Figure 2

## Slide 3
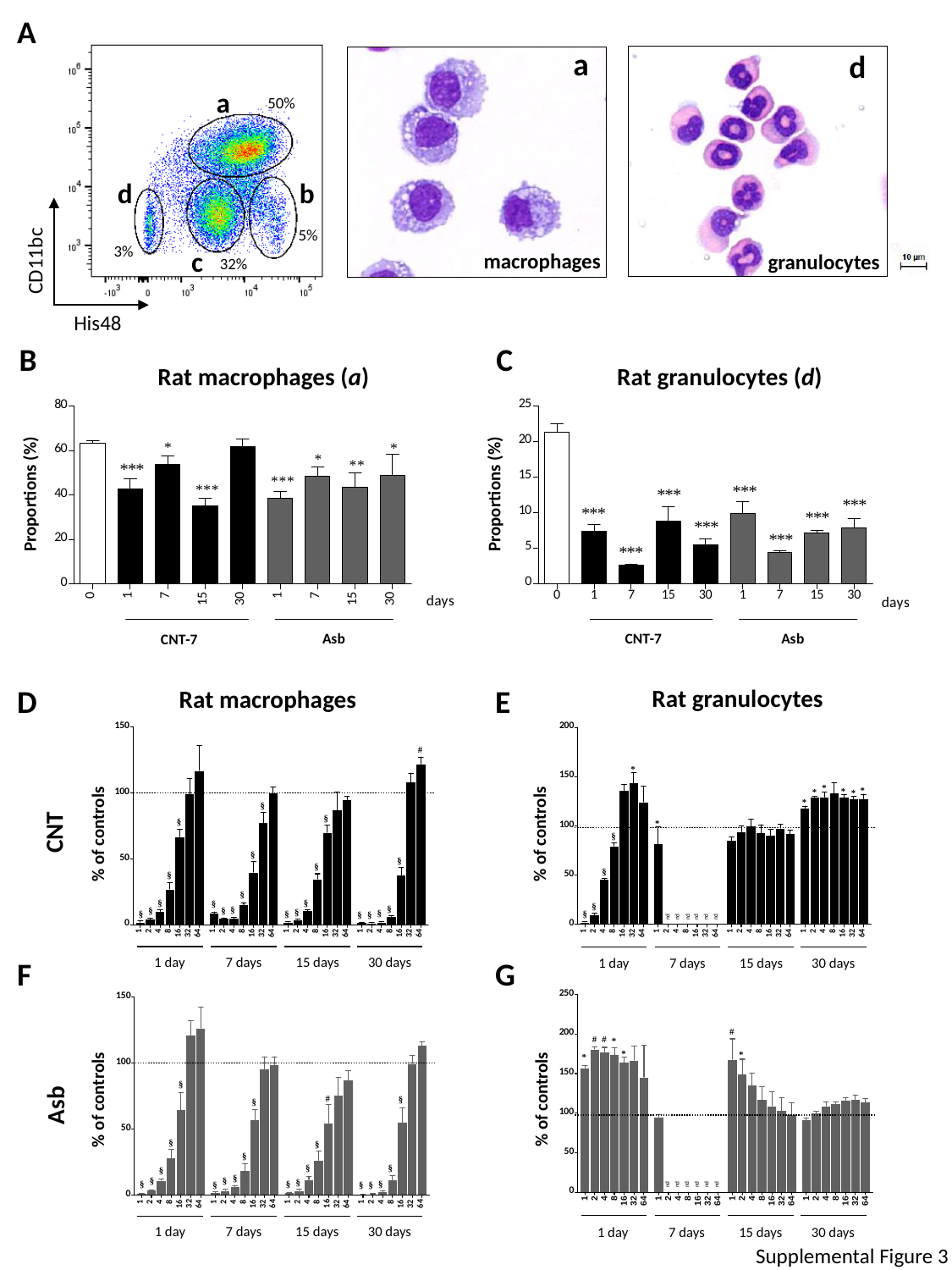

A
a
d
a
50%
d
b
CD11bc
His48
5%
3%
c
macrophages
granulocytes
32%
C
B
Rat macrophages (a)
Rat granulocytes (d)
Proportions (%)
Proportions (%)
D
E
Rat granulocytes
Rat macrophages
% of controls
1 day
7 days
15 days
30 days
% of controls
1 day
7 days
15 days
30 days
CNT
F
G
% of controls
1 day
7 days
15 days
30 days
% of controls
1 day
7 days
15 days
30 days
Asb
Supplemental Figure 3

## Slide 4
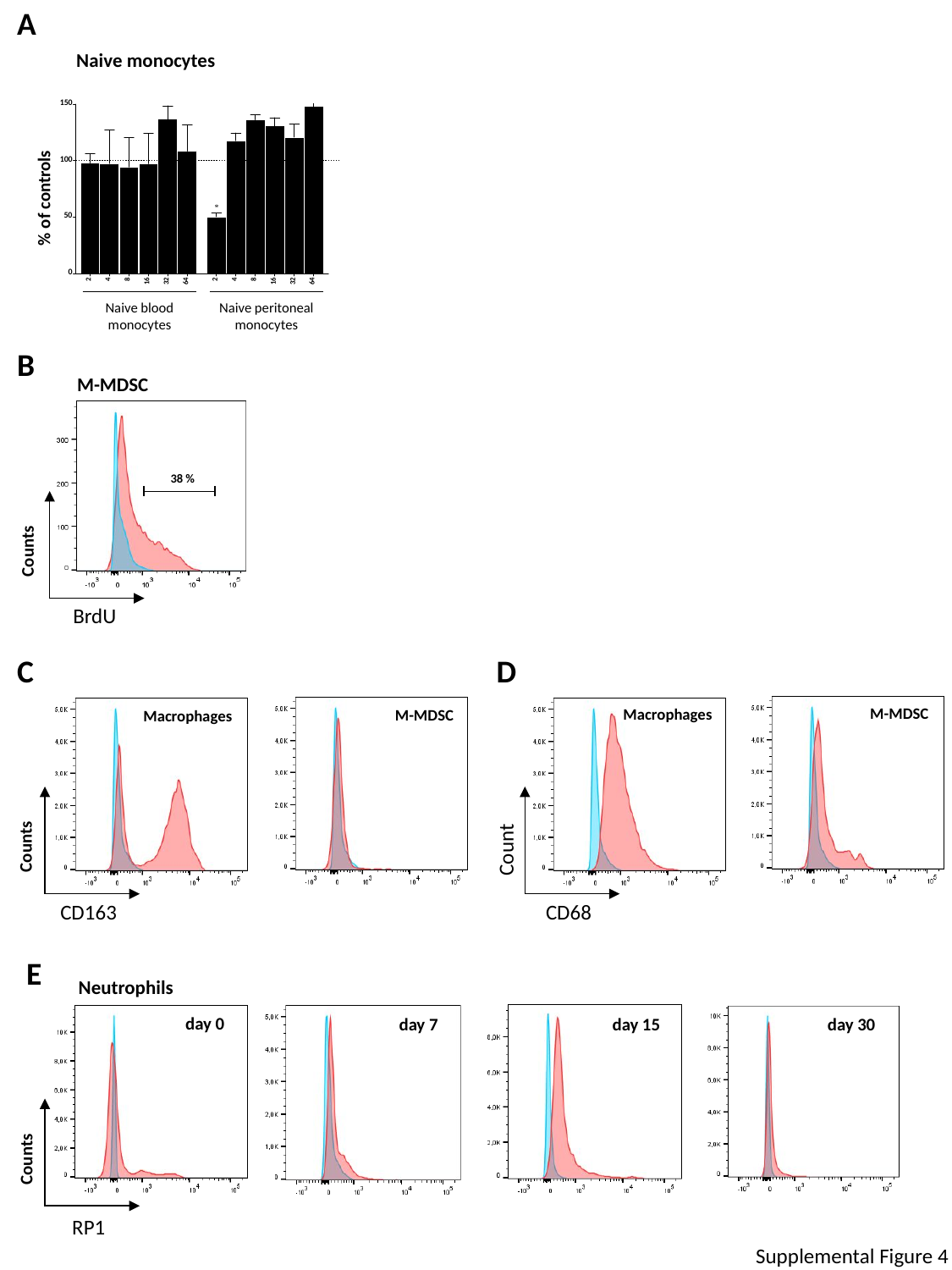

A
Naive monocytes
% of controls
Naive blood
monocytes
Naive peritoneal
monocytes
B
M-MDSC
38 %
Counts
BrdU
C
D
M-MDSC
Macrophages
M-MDSC
Macrophages
Counts
Count
CD68
CD163
E
Neutrophils
day 0
day 7
day 15
day 30
Counts
RP1
Supplemental Figure 4

## Slide 5
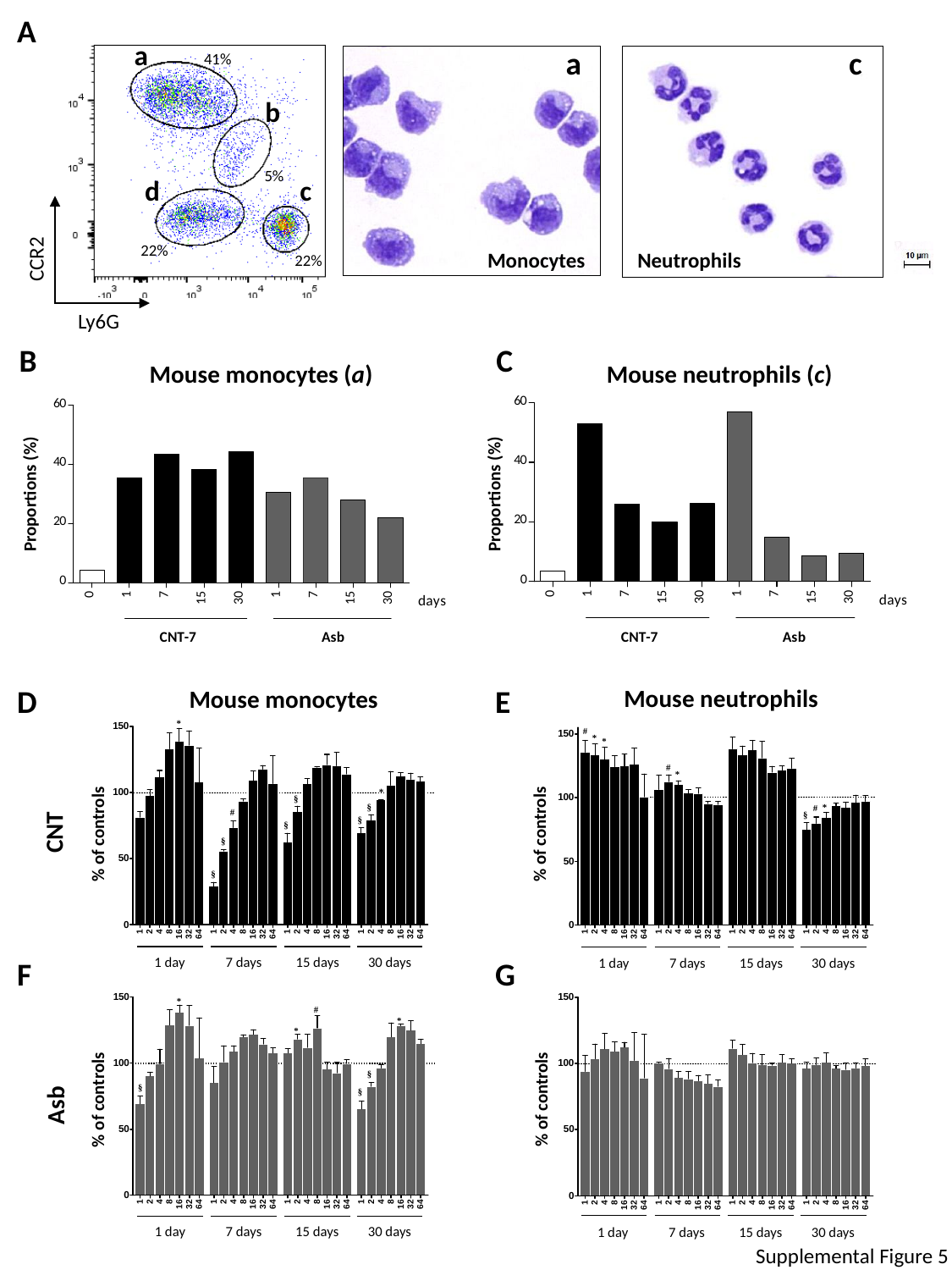

A
a
a
c
41%
b
5%
d
c
CCR2
Ly6G
22%
Monocytes
Neutrophils
22%
C
B
Mouse monocytes (a)
Mouse neutrophils (c)
D
E
Mouse neutrophils
Mouse monocytes
% of controls
1 day
7 days
15 days
30 days
CNT
% of controls
1 day
7 days
15 days
30 days
F
G
% of controls
% of controls
Asb
1 day
7 days
15 days
30 days
1 day
7 days
15 days
30 days
Proportions (%)
Proportions (%)
Supplemental Figure 5

## Slide 6
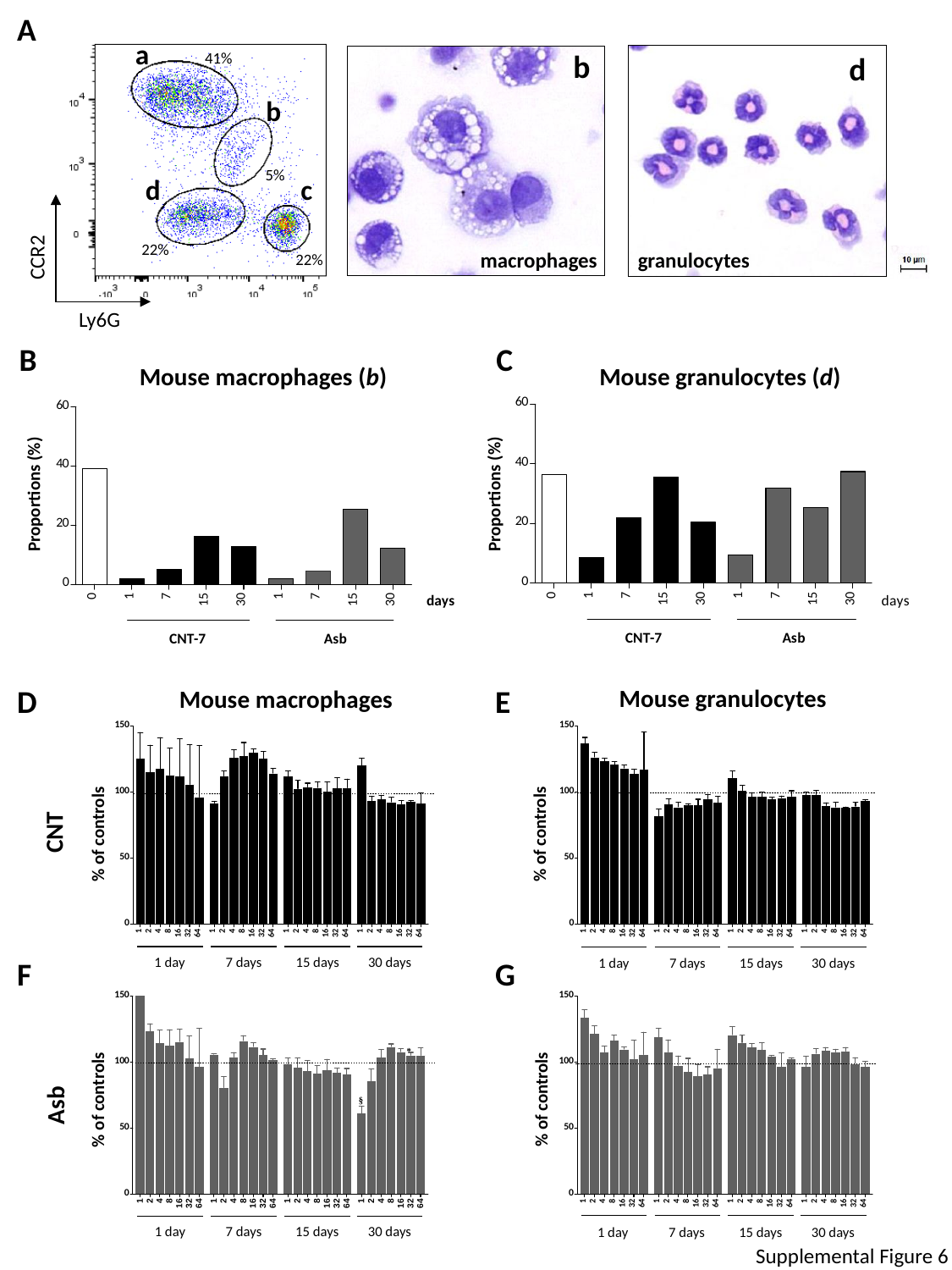

A
a
b
41%
d
b
5%
d
c
CCR2
Ly6G
22%
macrophages
granulocytes
22%
C
B
Mouse macrophages (b)
Mouse granulocytes (d)
D
E
Mouse granulocytes
Mouse macrophages
% of controls
1 day
7 days
15 days
30 days
% of controls
1 day
7 days
15 days
30 days
CNT
F
G
% of controls
1 day
7 days
15 days
30 days
% of controls
1 day
7 days
15 days
30 days
Asb
Supplemental Figure 6
Proportions (%)
Proportions (%)

## Slide 7
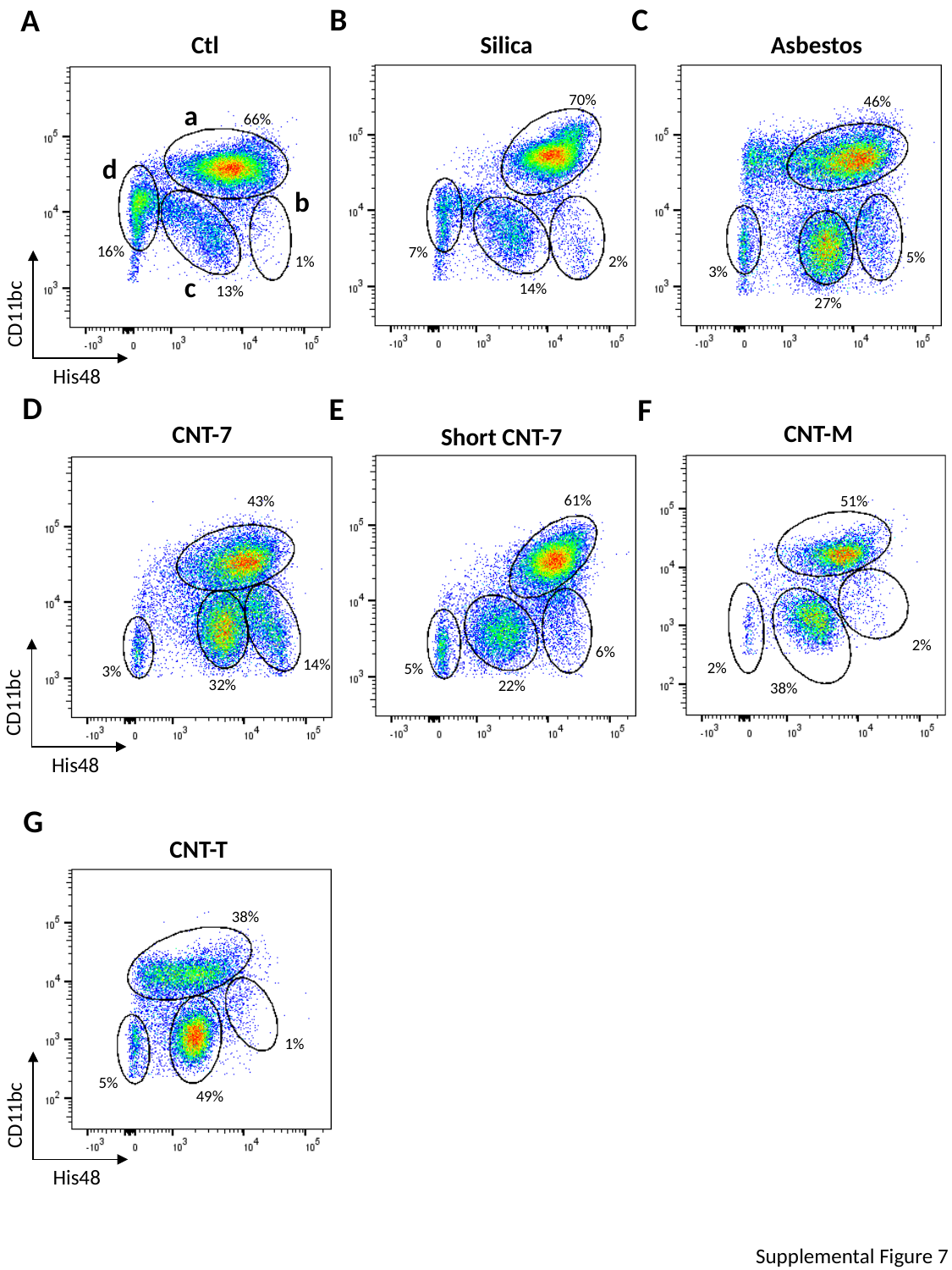

B
C
A
Ctl
Silica
Asbestos
70%
46%
a
66%
d
b
16%
7%
5%
1%
2%
3%
c
14%
13%
27%
CD11bc
His48
D
E
F
CNT-M
CNT-7
Short CNT-7
61%
43%
51%
2%
6%
14%
2%
5%
3%
32%
22%
38%
CD11bc
His48
G
CNT-T
38%
1%
5%
49%
CD11bc
His48
Supplemental Figure 7
