# Supplementary material for: Mesothelioma response to carbon nanotubes is associated with an early and selective accumulation of immunosuppressive monocytic cells
Source: Part Fibre Toxicol. 2016 Aug 23;13:46. doi: 10.1186/s12989-016-0158-0 (PMC4994252; doi:10.1186/s12989-016-0158-0)
Supplement: Supplementary file 2 — Characteristics of the carbon nanotubes. (DOCX 200 kb) [file 12989_2016_158_MOESM2_ESM.docx]

**Supplemental Table 1: Characteristics of the carbon nanotubes**

|  | CNT-7 | Short CNT-7 | CNT-M^19,41^ | CNT-T^17^ | crocidolite |
| --- | --- | --- | --- | --- | --- |
|  |  |  |  |  |  |
| Median length (µm) | 7.1 | 2.8 | 0.7 | 3 | 3 |
| Diameter (nm) | 75 | 75 | 11.3 | 15 | 200 |
| Fibres >5 µm (%) | 78 | 14 | <1 | n/a | 5 |
| Raman spectroscopy (D/G ratio) | 0.09 | 0.15 | 1.16 | 1.5 | n/a |
| Metallic impurities (%w)  Fe  Co  Ni  Mo | 0.35  <0.001  <0.001  <0.001 | 0.35  <0.001  <0.001  <0.001 | 0.48  0.49  <0.001  <0.001 | n/a | n/a |

Transmission EM (TEM) images for CNT-7 (**A**) and short CNT-7 (**B**).


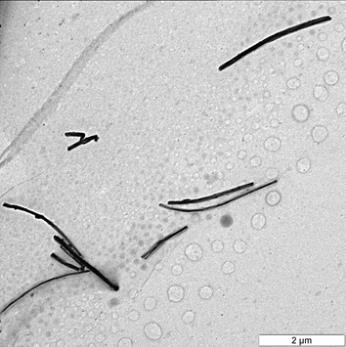

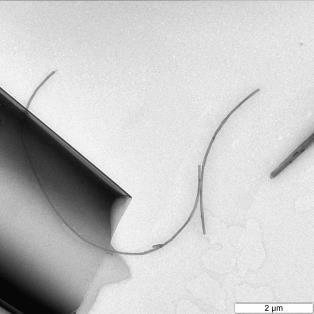


**B**

**A**
